# Foreword for neuroplasticity and neurorehabilitation

**DOI:** 10.3389/fnhum.2014.00544

**Published:** 2014-07-24

**Authors:** Edward Taub

**Affiliations:** Department of Psychology, University of Alabama at BirminghamBirmingham, AL, USA

**Keywords:** neuroplasticity, neurorehabilitation, central nervous system, rehabilitation, cortical reorganization

For much of the twentieth century, the views concerning the plasticity of the mature mammalian central nervous system (CNS) and the possibility of improvement in functional impairments in the chronic phase following substantial damage to the CNS were fixed and rarely questioned. The potential for plastic CNS change was thought to be confined to the immature organism, but in the adult the structure of the brain and spinal cord was believed to be hard-wired and unchanging no matter rehabilitation or environmental influence was applied after CNS damage. This belief was perhaps most prominently stated by Ramon y Cajal (1928), but its origin can be traced to very near the beginning of the scientific study of the nervous system, as embodied in the work of Louis Broca on the anatomic localization of motor function in the brain (Broca, 1861). Alternate views were periodically expressed (Fleurens, 1842; Fritsch and Hitzig, 1870; Munk, 1881; Lashley, 1938), but they were very much in the minority. An important experimental challenge to the standard view of the structural immutability of the CNS came in the discovery by Liu and Chambers (1958) of collateral intraspinal sprouting of dorsal root axons after spinal cord damage. Subsequent work by Goldberger (1977) and Goldberger and Murray (1974) confirmed this finding and subsequently it was demonstrated that sprouted fibers establish synaptic connections in the brain as well (Raisman, 1969; Raisman and Field, 1973; Tsukahara et al., 1975). These findings engendered considerable interest in terms of their potential relevance to recovery from spinal cord injury. However, for one-quarter century after the discovery of collateral intraspinal sprouting, there was no clear demonstration that its occurrence in the brain could have important significance for the motor or sensory function of an adult mammal. There was thus no compelling evidence-based reason for altering the prevailing view of an anatomically fixed brain.

In the field of rehabilitation, the older view concerning the limited amount of recovery of function that was possible in the chronic phase after CNS injury also had the status of axiomatic belief, which, if anything, was even more firmly held. There was a general opinion that rehabilitation treatment before the end of the spontaneous recovery period could accelerate its progress and perhaps even elevate its final level somewhat in the case of motor function, but there was no consensus for the latter belief and there was also no credible, controlled evidence that this was possible. In fact, powerful evidence to the contrary was part of the practice of every physiatrist, neurologist, and rehabilitation therapist. The process of spontaneous restitution of function was routinely observed to proceed with progressively decreasing speed until a plateau was reached, and subsequently additional recovery did not occur no matter what rehabilitation method was employed. This observation was universal and its implied principle of there being a barrier to improvement in function in the chronic phase after CNS injury was viewed as being self-evident.

The belief in the lack of potential for rehabilitative change long after CNS damage and the lack of plasticity in the mature mammalian brain, when these subjects were considered together at all, were thought to be reflections of one another. One implied and seemed to confirm the other. Hughling Jackson's hierarchical view that lower centers of the brain, capable only of providing the basis for impaired performance, substituted in function for higher damaged centers after CNS insult (Jackson, 1873, 1884) and other similar formulations powerfully influenced thought for most of the twentieth century. However, in the 1970s several investigators, Wall (Wall and Egger, 1971; Dostrovsky et al., 1976) among others, obtained findings that they interpreted as indicating that environmental influences, including training, could induce plastic change in the injured brain. These conclusions were preliminary. The experimental breakthrough came in the work of Merzenich (Merzenich et al., 1983, 1984; Jenkins et al., 1990) and Kaas et al. (1983) and their co-workers in the 1980s and early 1990s. There was a comparable development in the field of neurorehabilitation. In 1993 a paper (Taub et al., 1993), based on several decades of basic research on somatosensory deafferentation in old world monkeys (Taub, 1977, 1980), reported on a rehabilitation procedure, termed Constraint-Induced Movement therapy or CI therapy, that could produce substantial improvement in upper extremity motor function in humans many years after stroke. It was later found that CI therapy produced substantial functional (Liepert et al., 1998, 2000; Kopp et al., 1999) and structural (Gauthier et al., 2008) changes in the brain. These findings overturned the classic views on the unmodifiability of the CNS after damage and the unmodifiability of functional deficits persisting into the chronic phase after brain damage. The papers in this collection describe the subsequent findings in the field of neuroplasticity and neurorehabilitation. Many of the investigators who have made the key discoveries in these fields are the senior or co-authors of these papers. Persuasive evidence has been accumulating at a rapidly accelerating pace that the two areas are closely related. For the future, the newly developed areas of research based on these observations hold great promise for arriving at fundamental discoveries on the potential for plastic change in the damaged nervous system, how this can be produced by behavioral training, environmental influences, and other extrinsic manipulations, and how these procedures can be employed to effect much greater recovery of function of CNS damage than had previously been thought to be possible.

## Conflict of interest statement

The author declares that the research was conducted in the absence of any commercial or financial relationships that could be construed as a potential conflict of interest.
